# Editorial: Micro/nano materials for energy storage and conversion

**DOI:** 10.3389/fchem.2023.1150885

**Published:** 2023-03-07

**Authors:** Shiyong Zhao, Hailong Wang, Andreas Ruediger, Feng Gu, Dongliang Yan, Jinlin Lu

**Affiliations:** ^1^ Australian Carbon Materials Centre (A-CMC), School of Chemical Engineering, University of New South Wales, Kensington, NSW, Australia; ^2^ School of Materials and New Energy, Ningxia University, Yinchuan, China; ^3^ Nanoelectronics-Nanophotonics INRS-EMT, Université Du Québec, Québec, QC, Canada; ^4^ Laboratory of Advanced Materials and Manufacturing, Nanchang Key Laboratory for Advanced Manufacturing of Electronic Information Materials and Devices, International Institute for Innovation, Jiangxi University of Science and Technology, Nanchang, Jiangxi, China; ^5^ School of Materials and Environment, Guangxi Minzu University, Nanning, China; ^6^ Research Center for Corrosion and Erosion Process Control of Equipment and Material in Marine Harsh Environment, Guangzhou Maritime University, Guangzhou, Guangdong, China

**Keywords:** nanotechnology, micro/nano materials, energy conversion, catalysis, battery

The demand for energy is increasing dramatically at an alarming rate, resulting from rapid economic development and the ever-increasing requirements of energy-based appliances. With energy usage increasing, concerns about environmental Research Topic and the social problems associated with the consumption of conventional fossil fuels are becoming serious (Yu D. et al., 2014; Fang R. et al., 2017). As a cutting-edge approach, nanotechnology has opened new frontiers in the field of materials science and engineering to meet the challenge by designing novel materials, especially micronanometer, subnano, and even atomic scale materials, for efficient energy storage and conversion. Recently, the applications of micro/nano materials in energy storage and conversion fields, including lithium batteries, metal-ion batteries, water splitting, photocatalytic reactions, and electrochemical catalysis, have been widely investigated (Dai L. et al., 2015; Hao J. et al., 2020; Zhang S. et al., 2022). However, the practical application of micro/nano materials is still far from being satisfactory, as it is mainly impeded by costs and efficiency. Therefore, the design of cost-saving and highly efficient micro/nano materials in the field of energy storage and conversion is still very significant. Numerous papers have been reported in this Research Topic, and herein we introduce the representative advances in the collected papers that discuss how micro/nano materials work in the area of energy conversion and storage.

Currently, the highest energy density of lithium-ion batteries (LIBs) is approaching its limitation but is still unable to satisfy the growing requirements of electric vehicles. Furthermore, the high cost and safety Research Topic severely limit their large-scale practical application for renewable energy storage systems. It is urgent to develop an innovative and highly efficient battery system to meet the ever-increasing demands for energy. Cao et al. reported one electrospinning approach to synthesize a novel nanocompound, namely, the FeS_2_ nanoparticles encapsulated in S/N co-doped three-dimensional multi-channel structural carbon nanofibers (FeS_2_@ CNFs). The FeS_2_@ CNFs electrode exhibited an excellent rate property and cyclic stability as cathode materials for LIBs. The electrode also showed a high initial capacity of 1,336.7 mAh g^−1^, and it still had a capacity of 856.5 mAh g^−1^ remaining at 0.02A g^−1^ after 100 cycling tests. Li et al. fabricated a new ZnO@CZIF-8 nanocompound comprising the hierarchical ZnO nanospheres coated with inherently derived ZIF-8 porous carbon shells, which could provide sufficient active sites, facilitate rapid electronic migration, and effectively restrain the volume expansion of electrode materials. Finally, the ZnO@CZIF-8 nanospheres electrode exhibited a high capacity of 316 mAh g^−1^ at a current density of 1 A g^−1^ after 50 cycles and a satisfactory rate capacity used as the anode material for the Ni-Zn secondary battery using a commercial Ni(OH)_2_ cathode. Iron–chromium redox flow batteries (ICRFBs) possessed the advantages of long cycling performance, flexible design, and high safety, as well as affordable maintenance costs. Su et al. reported one method to prepare an indium ions composite electrode by introducing the indium ions into graphite felt surfaces, which exhibited dramatically enhanced electrochemical properties after In^3+^ modification.

It is worth noting that the aqueous metal-ion batteries with high safety and economical costs show promising opportunity for the development of an efficient large-scale energy storage system. Gan et al. summarized the main factors of the cyclic stability attenuation of cathode materials and the strategies of how to optimize the stability of cathode materials for aqueous zinc-ion batteries, including introducing vacancy, doping, combination engineering, and object modification. Besides these, the applicable material system and corresponding mechanisms of the relevant optimization strategies were provided, and finally, perspectives of further research directions and development prospects were proposed for practical industrial application. Sodium-ion batteries (SIBs) have attracted much attention as potential alternatives to LIBs owing to their high abundance, environmental friendliness, and low cost. Wang et al. reviewed the significant research progress on Sb_2_S_3_-based nanomaterials for SIB, mainly focusing on Sb_2_S_3_, Sb_2_S_3_/carbon composites, Sb_2_S_3_/graphene composites, and the Sb_2_S_3_/M_x_S_y_ composites structure. Sb_2_S_3_-based micro/nano materials displayed promising potential for developing high-performance SIBs.

Besides the batteries energy system, supercapacitors are a significant device because of their many advantages, such as high power density, good charge/discharge rate performance, and long cycling stability. Liu et al. synthesized a flower-like MnO_2_/G microsphere by optimizing the TE-G/KMnO_4_ ratio of carbon and KMnO_4_ in the redox reaction. The MnO_2_/G electrode demonstrated a superior rate performance with a specific capacitance of 500 F g^−1^ at the current density of 1 A g^−1^, and the capacitance retention was kept at 85.3% after 5,000 cycles tests, which was much better than the optimized MnO_2_/rGO electrode. Wu et al. reported a hybrid Ti_3_C_2_Tx/C nanosphere microsupercapacitor structure through aerosol jet printing technology. The planar devices were manufactured by the hybrid spherical nanostructures, which showed excellent areal capacitance performance. This design provided a straightforward and effective technique on how to build up a 3D-structured MXene with suppressed self-stacking in order to achieve microenergy storage devices with high electrochemical performances.

Moreover, photocatalysis technology has attracted wide attention recently due to its good performance in degrading series of toxic compounds. It can provide an efficient approach to solve environmental problems. Lu et al. synthesized a ZnO-reduced graphene oxide (rGO) solid catalyst through a one-step electrodeposition method, with lithium perchlorate (LiClO_4_) as the supporting electrolyte on the FTO substrate. Thanks to the cooperative effect between rGO and ZnO, the as-obtained ZnO-rGO structure showed a much-enhanced photocatalytic degradation performance. The degradation rate of methylene blue could reach up to 99.1% in 2 h through optimizing the ZnO-rGO composite structure by adjusting the electrodeposition process, which confirmed the effectiveness of the hierarchical approach. Yin et al. fabricated periodic epitaxial junctions utilizing Sb_2_Te_3_ nanoblades serialized by Te nanowires (Sb_2_Te_3_/Te) through a one-step hydrothermal epitaxial growth method. The as-obtained product possessed a good crystal shape and heterojunction construction, resulting in a very fast photo response owing to the efficient separation of photogenerated carriers. The responsivity and detectivity were 9.5 × 10^11^ μAW^−1^ and 1.22 × 10^11^ Jones at 50 K, respectively, thus exhibiting a better detection ability than other Te-based photodetector devices.

Micro/nano materials also play a significant role in the field of electrocatalysis. Zhang et al. reported one facile organic–inorganic hybridization approach to synthesize Co-N-C_X_ catalysts, which showed excellent hydrogen evolution reaction (HER) performances, achieving a low overpotential of 145 mV to reach 10 mA cm^−2^ in 0.5 M sulfuric acid. This Co-N-C catalyst greatly facilitated the charge transfer to enhance the HER kinetics, and it also improved the durability during the long cycling tests. Cao et al. reported a feasible molecular self-assembly method to fabricate Pt/Mo carbide/multi-walled carbon nanotubes (Pt/MoCx/MWCNTs) as an active electrode for ethanol electrooxidation reaction (EOR) in acid media. The composite catalyst demonstrated high catalytic activity and a prominent anti-CO poisoning ability. As described in the paper, the abundant exposure of the active sites and the synergistic effect between Pt and MoC contributed to the superior EOR performance.

Besides experimental methods, simulation and numerical analysis are very powerful tools to design and develop a novel energy storage and conversion system. Fahim et al. reported a numerical analysis that was performed to enhance the heat transmission in the receiver of a parabolic solar collector by introducing perforated barriers. In this work, the flow and thermal characteristics of a solar collector were investigated. Moreover, it also analyzed the beneficial effects of using perforated baffles to improve the heat transfer. The position and perforation number were optimized to achieve the best heat transfer. How to achieve efficient heat transfer and energy storage is still a key problem for engineers and industrialists. Adnan et al. studied the energy storage efficiency between (Al_2_O_3_-CuO-Cu/H_2_O)_mhnf_ and (Al_2_O_3_-CuO/H_2_O)_hnf_ under the condition of novel viscous dissipation effects. The results confirmed that the third generation of heat transfer fluids (Al_2_O_3_-CuO-Cu/H_2_O)_mhnf_ possessed a much higher thermal energy storage efficiency than that of the traditional nano and hybrid nanofluids. Overall, the new insights in heat transfer are promising and could help deal with the requirements of energy storage that must be met in the modern technological world.

We sincerely hope that this Research Topic will inspire and provide new ideas for the design and fabrication of novel micro/nano materials for energy storage and conversion. All the collected works have contributed significantly to novel micro/nano materials design and synthesis. Furthermore, all the manuscripts have innovatively provided new approaches for the field of energy storage and conversion, covering experimental and theoretical calculations. Moreover, some suggestions have been provided with respect to the development of the field of energy conversion and storage. The realization of a heterostructure is a significant and promising means to improve the performance of micronanostructures in energy storage and conversion; this deserves more research efforts. This heterogeneous structure can achieve a good synergistic effect, combining different performance advantages so as to enhance the overall performance and open up different applications in different fields. Finally, we sincerely thank all the authors, reviewers, and editors who have highly contributed to this Research Topic.
